# Help-seeking in emerging adults with and without a history of mental health referral: a qualitative study

**DOI:** 10.1186/s13104-016-2227-8

**Published:** 2016-08-24

**Authors:** Ruth Spence, Matthew Owens-Solari, Ian Goodyer

**Affiliations:** 1 Centre for Abuse and Trauma Studies, Middlesex University, The Burroughs, London, NW4 4BT UK; 2 Mood Disorders Research, University of Exeter, Exeter, UK; 3 Developmental Psychiatry, Cambridge University, Cambridge, UK

**Keywords:** Emerging adults, Help-seeking, Qualitative research, Mental health, Stigma

## Abstract

**Background:**

Young people are generally reluctant to seek professional help when experiencing problems. However, past experience of services is often cited as increasing the intention to seek help, therefore those with a history of mental health referral may adopt more adaptive help seeking strategies. The current study investigated whether the pattern of different help seeking strategies and barriers to help seeking differed as a function of previous referral history.

**Methods:**

Semi-structured interviews were conducted with 29 emerging adults (12 males, 17 females); 17 with a history of mental health referral and 12 without and analysed using thematic analysis.

**Results:**

Overall, those with a referral to services were more likely than those without to rely on avoidant coping, especially techniques that depended upon suppression. This could help account for the increased use of strategies involving self-harm and substances in those with past referral. An exploration of barriers to help seeking showed those with a history of mental health referral were much more likely to self-stigmatise and this became attached to their sense of identity.

**Conclusions:**

Emerging adults with a history of referral are more likely to adopt avoidant coping strategies when dealing with problems and self-stigmatise to a greater degree than those without a history of referral. This suggests that current approaches to mental health in emerging adults are not decreasing the sense of stigma with potentially far-reaching consequences for the developing sense of self and choice of help seeking strategies.

## Background

Over recent years, child and adolescent mental health services have seen a decrease in mental health spending set against an increasing demand for services [1, 2]. Indeed, the prevalence rates of clinical diagnoses increases during adolescence and emerging adulthood [3] and young people are particularly susceptible to common mental health problems like anxiety and depression [4]. Therefore, it is particularly important that young people both want to and are able to access professional help if needed.

Certainly, it is also at this time when individuals become responsible for their own health behaviours [5]. One such example is the use of ‘avoidant’ or ‘approach’ coping strategies. Whereas avoidant coping involves attempts to escape from stressors or deny their existence [6], help seeking is an adaptive approach form of coping. It includes actively engaging with the problem and requesting assistance from others, and there is a large literature suggesting actively coping with problems leads to better adjustment across a range of contexts [e.g. 7–10] whereas avoidant coping is associated with lower satisfaction and greater clinical symptoms [e.g. 11–13].

Nevertheless, young people are generally reluctant to seek professional help and recently there has been a drive to increase the parity of esteem for mental health in the UK [14]. Commonly cited barriers to help seeking include a preference for self-reliance and stigma [15–17], whereas positive past experiences of services are often considered to increase the intention to seek help [15, 18, 19]. This suggests those with a history of mental health referral may adopt more adaptive help seeking strategies when faced with problems.

Any differences in patterns of help seeking may be particularly noticeable when seeking help from friends and family as young adults are more likely to use these sources when experiencing problems [16] with parents and peers acting as gatekeepers, encouraging those who are distressed to access appropriate professional support. Indeed, research suggests that the majority of men who access professional psychological services are influenced by others to do so [20] and parents are a common influence on adolescents in regard to seeking help from mental health services [16].

The current study explored help-seeking in young adults with different histories of mental health referral and reports on patterns of help-seeking strategies as a function of referral history; and any differences between groups on the barriers that prevent help-seeking from informal networks.

## Methods

### Participants

Participants are part of the ongoing ROOTS project; a longitudinal study of 1208 participants (547 males, 661 females) recruited from Cambridgeshire schools in the UK between April 2005 and December 2006 when they were aged 14 [4]. During the ROOTS study we asked each participant about their history of mental health referral, based on this we grouped participants as either having had or not had a mental health referral. The sample was stratified based on gender and history of referral; and a sub-sample of 29 participants (12 males, 17 females) were chosen at random to be interviewed using the ROOTS Help-Seeking Interview.

The majority of participants with a referral had sought help for problems with depression or anxiety, although four participants had seen professionals for problems not directly related to mental health (e.g. parents’ divorce or bereavement), the history of service use associated with a referral ranged from talking to a GP once to seeing a psychiatrist over a number of years. Two participants without a referral talked about experiencing problems where they considered seeking professional help (see Table 1). Although the referred group had a history of defined mental health problems whereas the non-referred group did not (itself a possible effect of contact with mental health services), every respondent was able to talk about having experienced problems and seeking help or otherwise when facing them. Equally, there was no significant difference between the groups on mood or anxiety symptoms (see below) when measured alongside history of referral at age 17.5 (see Table 1) suggesting some comparability between the two groups.

**Table 1:** Participant characteristics across history of referral

Gender		Problem type					Symptoms	
M	F	Mood/anxiety	Behaviour/anger	Family/relationship	Other	None	MFQ^a^	RCMAS^a^
Referral								
7	10	10	3	3	1	0	6.4	15.7
No referral								
5	7	1	0	1	0	10	5.5	11.1

^a^Average MFQ and RCMAS scores for referred and non-referred groups at age 17.5, differences are not statistically different (*p* > 0.05)

Respondents were aged between 20.1 and 22.2 (mean age 21.2, SD = 0.49). There were 17 (58.6 %) emerging adults with a history of mental health referral (7 males, 10 females). The study was approved by a National Health Service Research Ethics Committee prior to commencement and all participants gave informed consent.

### Measures

#### History of mental health referral

As part of the ROOTS study we recorded each participant’s lifetime history of mental health referral. At age 14.5, we used a self-report questionnaire and recorded a binary yes or no response. At age 17.5, we gathered information regarding lifetime history of referral to any mental health service, including their GP, and any subsequent service use from the participants through a combination of a Health Services Interview and answers to the Kessler Psychological Distress Scale [K10; 21]. The K10 has demonstrated its validity as a marker of general distress among individuals in the general population [22]. It is a ten item self-report questionnaire which allows individuals to rate themselves for symptoms of depression (e.g. feeling tired, feelings of worthlessness) and anxiety (e.g. feelings of nervousness, feeling restless) over the previous 4 weeks. We asked participants whether they had gone to see their GP because of these feelings. If participants had missing K10 information but stated during the interview that they had never been referred to services, we recorded them as having no history of referral.

#### The ROOTS help-seeking interview

We developed the semi-structured ROOTS Help-Seeking Interview especially for the study to explore the different help-seeking strategies used by the respondents and barriers to seeking help in emerging adults. Any individual who reported they had never been referred to services were asked about help-seeking and coping strategies used when they faced problems or stress [e.g. *“When you have a personal problem, do you try doing anything to make yourself feel better?”, “Do you talk to your friends about it? (why not?)”*]. Those with a referral were asked about help-seeking and coping strategies they had used leading up to their referral [e.g. *“Before going to see someone, was there anything you tried doing first to feel better?”, “Did you talk to your parents about it? (why not?)”*]. Audiotaped interviews took place over the telephone; interviews lasted on average 25 min and were flexible, because we omitted, adapted or elaborated questions in line with individual contexts.

#### Mood and feelings questionnaire (MFQ; [23])

The MFQ rates symptoms of depression based on DSM criteria, it is a widely used questionnaire with good validity in clinical and non-clinical child and adolescent populations [24]. Participants completed this 33 item self-report questionnaire at age 17.5. Descriptive phrases regarding how the subject has been feeling or acting over the last 2 weeks were rated on a Likert scale from 0 *“never”* to 3 *“always”*.

#### Revised children’s manifest anxiety scale (RCMAS; [25])

The RCMAS has been subjected to extensive study ensuring it is psychometrically sound for clinical and research purposes [26]. It is a self-report questionnaire designed to assess the level and nature of anxiety in children and adolescents aged 6 to 18 years, participants completed it at age 17.5. It contains 37 anxiety items that are answered on a four point scale from 0 “*never*” to 3 “*always*”. Only total anxiety was of interest in the present study, this is produced by summing 28 of the items.

### Analysis

We conducted the qualitative analysis in Atlas [27] using Thematic Analysis (TA) to provide a rich, descriptive account of how emerging adults view various aspects of service use. TA involves identifying, analyzing and reporting on themes found within the data. TA has the advantage of being a flexible technique that is suitable for use with any qualitative data [28] and is not aligned with a particular theory, but is rather led by the data [29]. Researchers can characterize TA as a technique to use within other approaches rather than a method of analysis; however, Braun and Clarke [30] argue TA is a method in its own right. This is because it lacks theoretical assumptions and therefore researchers can use it more flexibility across different approaches.

We conducted an inductive analysis where we coded the data without trying to fit it within a pre-existing coding frame or theoretical framework. We considered themes key if they were prevalent, (i.e. a number of different participants articulated a thought relevant to the theme). The analysis followed Braun and Clarke’s six phases of analysis: (a) familiarization; reading and re-reading the data, noting down initial ideas (b) generating initial codes; systematically coding interesting features of the data across the entire data set and collating data relevant to each code (c) searching for themes; collating codes into potential themes (d) reviewing themes; checking the themes work in relation to the coded extracts and the entire data set (e) defining and naming themes; refining the specifics of each theme to generate clear definitions and names (f) producing the report; selecting extract examples, relating the analysis back to the research question [30].

We identified initial codes from reading the transcribed interviews; we checked codes for emerging patterns and grouped then together into potential themes. We interpreted the themes by reading and re-reading the coded data extracts to ensure the themes were coherent. Themes were then refined accordingly to ensure consistency of the data extracts and codes contained within them. We chose key extracts to illustrate each theme and help with describing different aspects of help-seeking.

To determine the frequency and representativeness of responses we used the conventions outlined by Hill et al. [31]. A *general* response applies to all cases, *typical* cases apply to at least half of the cases and *variant* to at least two or three cases but less than half.

## Results

The participants mentioned numerous coping strategies throughout the interviews and most people used several. It appeared that strategy selection was not consistent and to some extent depended on individual context. This sometimes led to conflicting information, for example, someone might disclose that they went to see their GP after talking to a family member while also maintaining that they did not talk to others about their problem. Additionally, beliefs did not always match up with behaviours, for instance although everyone agreed that talking to others was beneficial some individuals were more reluctant to engage in this behaviour than others. Nevertheless, individuals seemed to present themselves as favouring specific coping mechanisms, in that they mentioned strategies with more or less frequency across the interviews.

### Pattern of coping mechanisms in referred and non-referred participants

Five main coping mechanisms were described across the interviews. We divided these into approach and avoidant strategies. Approach coping entailed engaging with the problem through seeking out other people for input “not just personal things, but like school and everything, I find it really important, I’m quite an independent person but I do find it really important to talk through things with somebody” (ID 1200, female, no referral). It also comprised strategies where the individual used techniques such as reflection and problem-solving to actively engage with their problem “if there was something playing on my mind I would just try and think it through and think rationally and logically, try and solve any problems that I had” (ID 1018, male, no referral).

Typically the participants used approach coping mechanisms when dealing with problems. More specifically, it was typical for respondents to talk about using others for support, whilst talking about problem solving was mentioned with variant frequency. Of those who used approach coping, respondents with a history of referral to services showed a preference for problem solving techniques and were less likely to use others as a source of support when compared to those without a referral (see Table 2).

**Table 2:** Counts of those using different help-seeking strategies

	Referred			Not referred		
	Males (n = 7)	Females (n = 10)	Total (n = 17)	Males (n = 5)	Females (n = 7)	Total (n = 12)
Talk	4	6	*10*	5	7	*12*
Problem solving	1	7	*8*	2	1	*3*
*Approach coping*	*5*	*13*	*18*	*7*	*8*	*15*
Ignore	6	8	*14*	1	1	*2*
Distraction	1	2	*3*	5	5	*10*
Destructive	3	4	*7*	1	0	*1*
*Avoidant coping*	*10*	*14*	*24*	*7*	*6*	*13*

Total number of individuals using each type of help-seeking strategy have been italicised

Participants were coded with multiple types of coping therefore counts can exceed sample size

A common avoidant strategy was distraction, which was mentioned with variant frequency. It involved the individual engaging in activities designed to take their mind off the problem “go and do something else, watch some TV. Generally just do an activity that doesn’t involve kind of, that, those people or doing the activity that is causing the stress in the first place” (ID 1162, male, no referral). The most common avoidant coping technique which was mentioned with typical frequency was ignoring the problem. Although similar to distraction, ignoring the problem had suppression at its root whereas distraction used diversion “when I was like 15–16 I thought oh yeah that’s, I guess that’s right, I’ll just try and forget about it and then it will go away” (ID 1003, female, referral). The participants also used self-destructive coping strategies, which were mentioned with variant frequency and were considered to be damaging in some way. These included self-harm or substance use “it was the self-harming, because like I’d want to hit out on other people but rather than doing it to them I did it to myself. It just, it felt better than actually talking to somebody about it” (ID 1400, female, referral). Participants with a referral were more likely to talk about ignoring problems and using self-destructive techniques but were less likely to distract themselves than those without a referral.

These results suggest regardless of their history of referral emerging adults use a mixture of approach and avoidant strategies; most commonly talking to others, problem solving, ignoring the problem, distraction or destructive coping. The most striking difference was in the choice of avoidant strategies with a greater use of ignoring problems amongst those who had a referral to services. The thoughts and feelings associated with different coping strategies were explored more in-depth using thematic analysis to create themes around barriers to help-seeking and compare these across the groups.

### Themes around barriers to help-seeking

#### Stigma

The majority of participants mentioned stigma. However, it appeared that there were two different forms. There was stigma associated with the attitudes of others and participants openly discussed this. Indeed, it was typical to mention being concerned about other people judging them when admitting to having problems “it’s always at the back of your mind. You always think ‘oh god’ kind of thing, you don’t want people to think bad of you, or you think maybe weird things or think that you’re strange” (ID 1111, female, no referral).

There was also stigma that came from within, which did not appear to be explicitly recognised. The respondents talked about using others to gauge what was normal and needing help was seen as a marker of being abnormal, “people look around, especially during teenage years, at other people and how they are coping or appear to be coping and if someone isn’t in line with what everyone else is doing then I think they feel like they’re not normal” (ID 2115, female, no referral). The need to fit in was associated with avoidant help-seeking behaviours, because individuals strove to hide their problems and made unfavourable comparisons between themselves and others on their perceived ability to cope “You feel a bit mental and you don’t really understand why and no-one else feels like that so you’re like why do I feel like that?” (ID 1003, female, referral).

I did not really want people to know that I was going to see a psychiatrist. I tried to hide it from my friends at school… I just did not want them to know that I was struggling… you are trying to make such a good impression as it is, you do not want to add something that could make you seem sort of, you know, weirder (ID 1445, female, referral).

This process of self-stigmatization appeared to become attached to their sense of identity, as could be seen in the use of derogatory words like “crazy” or “nutcase” to describe themselves. “I mean I didn’t take any pills when I was depressed and now I’m normal” (ID 1400, female, referral). “I think if you’re insane then you don’t really have the ability to check yourself in because you’re crazy and you don’t really know what’s going on” (ID 1514, female, referral). Self-stigma was much more prevalent amongst those who had a referral; 12 participants referred to problems in a disparaging way, 10 of whom had been given a mental health diagnosis, “I’ll just call it my crazy…. I’m officially medically a little bit unstable, which I find fun. I’ve decided to think of that as fun, just because that’s easier for me, oh god I’m insane” (ID 1261, male, referral). Additionally, there was sense of shame attached to having to have sought help “no, I didn’t talk to them about it… at the time you don’t want people to think you’re a failure” (ID 1001, male, referral).

#### Minimization

There was also a tendency for participants to minimize or try not to acknowledge any problems they did experience “I didn’t want to dignify them with a response I suppose and I just didn’t want to act upon them in any way that really acknowledged them” (ID 1586, male, referral). Minimization appeared to be a defence mechanism and was a typical response for those with a history of referral, whilst it was a variant response for those without. It enabled young people to downplay their problems and rationalize not actively dealing with it or approaching others for help, “to me it seemed like other people would have bigger problems and mine weren’t really worth nothing, yeah mine really weren’t worth talking about” (ID 1520, male, referral). It could also help individuals normalize any problems they did have, thus avoiding the need for self-stigmatization and giving them a reason not to approach others. Participants often expressed minimization through beliefs that their problems were not important enough to talk to others about “it’s like everyone’s got problems anyway so what makes me any different” (ID 1375, male, no referral).

In fact, even when the participants actively sought help from others there was a sense of minimizing the problem. Indeed, it appeared that the ability to avoid talking about any problem in detail seemed to be at the root of several perceived advantages of talking to friends or family. There was a belief that if people knew you, less would have to be divulged making the process of ‘discussing’ the problem easier, “they also know me so well you know it’s kind of, you don’t have to explain as much they can tell what I’m thinking without having to say it a lot of the time” (ID 1564, female, referral). Indeed, participants often cited a lack of an implicit understanding as a disadvantage of talking to others or going to a professional.

#### Self-reliance

Throughout all the interviews, regardless of referral history, there was a pervasive sense that the young people wanted to cope with problems by themselves “it wasn’t even so much that I minded telling them what was wrong, it was that it was hugely to do with, I thought it was stuff that I should be able to deal with” (ID 1586, male, referral). Certainly, the idea of being self-reliant was a potent barrier to help seeking and promoted insular behaviours “it’s something that I feel like I just have to deal with myself, I’m sure there’s people you can speak to, but I’d just rather not” (ID 1375, male, no referral).

#### Burden

The participants often presented their self-reliance as a way to protect others “I don’t really like [them] worrying about something that they don’t need to worry about because I can handle it” (ID 1375, male, no referral). Commonly, it was suggested that disclosing problems would in fact be burdening others and this attribution of burden therefore acted as a justification for avoidant coping “I wouldn’t like to worry my parents, I think they um, I think they’re the type of people who get worried easily, particularly my mum gets worried easily about these sorts of things so I prefer not to talk about that” (ID 1202, female, no referral).

## Discussion

Most emerging adults used a combination of approach and avoidant coping strategies when dealing with problems. It is possible that for many emerging adults, help seeking is a dynamic process that fluctuates between approach and avoidant coping as individuals struggle to identify symptoms, recognise problems and use others to manage distress and help interpret meaning [32, 33]. Overall, those who had a history of referral had a greater reliance on avoidant coping strategies, particularly methods associated with suppression. This may in part also explain the increased dependence on self-destructive coping mechanisms in this group. Whereas distraction can reduce distress [34], suppression is associated with the opposite effect [35]. Therefore substances and self-harm may be used to help regulate their emotional experience [36, 37].

An exploration of the barriers to using approach coping revealed that regardless of their history of referral the emerging adults felt they should not rely on others to help with their problems, considered talking with others as burdening them and minimised their problems. The use of minimisation could be a particularly problematic barrier for young people who need higher levels of support. Problem recognition among young people tends to be low [38]. Therefore, trying to downplay or normalize difficulties while simultaneously depending on others to provide adequate support could lead to many issues going undetected or worsening over time.

Despite current UK policies regarding parity of esteem for mental health [14] stigma was an important barrier to help seeking; however this differed by history of mental health referral. Indeed, it appeared that having sought help was associated with a sense of personal weakness across all the emerging adults and this seemed to become attached to individual identity. However, in those who had had a referral to services it appeared more likely to create an enduring sense of not being ‘normal’ as demonstrated through their greater use of derogatory terminology when talking about their problems. This suggests that stigma around mental health problems is not being effectively tackled, which is a potent barrier to seeking help and may prevent earlier engagement with services [16].

Previous studies have found that self-stigma is associated with negative attitudes towards treatment-seeking and negatively impacts the decision to seek information and help [13, 39, 40]. The attachment of self-stigma to developing identity in emerging adults may be of particular importance as this is the life-stage where individuals become more concerned with questions surrounding their identity and how they fit in [41, 42]. A sense of belonging helps legitimise the developing sense of identity [43] and defining themselves as ‘being different’ may detrimentally affect their sense of well-being [44]. Furthermore, in contrast to studies that have found a greater intention to seek help amongst those who have used services [15], our results suggest this group exhibit a greater use of avoidant coping, which may play a role in preventing future help seeking, potentially leading to poorer long term outcomes [45, 46]. This appears to reflect a disconnect between future plans and actual behaviour when experiencing a problem.

The current research was exploratory and was not designed to investigate causation; for instance, we did not specifically address why there are help seeking differences between those with and without a history of referral. It is possible the difference in coping strategies reflects the difference in symptomatology. Indeed studies find that adolescents that are suffering from some form of psychopathology are more likely to adopt avoidance strategies than their peers and that this might be a protective response to more chronic or severe stress [47]. However, the participants did not significantly differ on mood or anxiety symptoms when they were measured at age 17.5, suggesting that referral history is not purely associated with problem severity. Indeed, all the participants without a referral were able to talk about times where they had faced problems. Prospective longitudinal research could answer questions around how help-seeking strategy choice develops and if there are changes associated with the development of psychopathology. Further research is also needed to differentiate between the effects of psychopathology and referral on stigma.

## Conclusions

Emerging adults with a history of mental health problems and referrals appear to suffer from more rather than less self-stigma, which could lead them to rely on avoidant coping strategies when experiencing problems. Although there has been an increased focus on parity of esteem for mental health, more needs to be done to tackle stigma effectively in adolescents and emerging adults in order to encourage more adaptive help-seeking strategies. This could result in lowering the reliance on unhelpful strategies such as self-harm and substances and better long term outcomes.
